# Intranasal Administration for Pain: Oxytocin and Other Polypeptides

**DOI:** 10.3390/pharmaceutics13071088

**Published:** 2021-07-16

**Authors:** Vimala N. Bharadwaj, Alexander Z. Tzabazis, Michael Klukinov, Neil A. Manering, David C. Yeomans

**Affiliations:** 1 Department of Anesthesiology, Perioperative and Pain Medicine, School of Medicine, Stanford University, Stanford, CA 94305, USA; vimalanb@stanford.edu (V.N.B.); klukinov@stanford.edu (M.K.); 2 Department of Anesthesiology and Critical Care, Campus Lübeck, University Hospital Schleswig-Holstein, 23538 Lübeck, Germany; alexander.tzabazis@uksh.de; 3 PIH Health, Department of Neurology, Santa Fe Springs, CA 90670, USA; nmanering@gmail.com

**Keywords:** intranasal delivery, pain, craniofacial pain, oxytocin, polypeptides, trigeminal system, nasal–cerebral pathway, migraine, nasal administration, neuralgia

## Abstract

Pain, particularly chronic pain, remains one of the most debilitating and difficult-to-treat conditions in medicine. Chronic pain is difficult to treat, in part because it is associated with plastic changes in the peripheral and central nervous systems. Polypeptides are linear organic polymers that are highly selective molecules for neurotransmitter and other nervous system receptors sites, including those associated with pain and analgesia, and so have tremendous potential in pain therapeutics. However, delivery of polypeptides to the nervous system is largely limited due to rapid degradation within the peripheral circulation as well as the blood–brain barrier. One strategy that has been shown to be successful in nervous system deposition of polypeptides is intranasal (IN) delivery. In this narrative review, we discuss the delivery of polypeptides to the peripheral and central nervous systems following IN administration. We briefly discuss the mechanism of delivery via the nasal–cerebral pathway. We review recent studies that demonstrate that polypeptides such as oxytocin, delivered IN, not only reach key pain-modulating regions in the nervous system but, in doing so, evoke significant analgesic effects. IN administration of polypeptides has tremendous potential to provide a non-invasive, rapid and effective method of delivery to the nervous system for chronic pain treatment and management.

## 1. Introduction

Recent developments in biochemistry and molecular biology have contributed to improved understanding of polypeptides as key signal transmitters in the central nervous system [1,2]. Polypeptides such as oxytocin are linear organic polymers consisting of two or more amino acids. The use of peptides as pharmacological agents is attractive due to low toxicity of their metabolites and strong potency [3,4,5]. Although peptides show potential to treat neurological diseases and disorders, they are largely limited as pharmaceuticals for treatments due to the inadequate deposition of functional peptides to specific brain regions. Under physiological conditions, peptide delivery to the brain is limited by the presence of the blood–brain barrier (BBB), which inhibits most therapeutic peptides from entering the brain from blood [6]. In addition, peptides administered orally have generally poor bioavailability and short half-lives due to enzymatic metabolism [7,8]. Parenteral administration routes, such as intravenous, subcutaneous or intramuscular injections, often cannot reach meaningful effect-site concentrations within the central nervous system secondary to the BBB. Although brain-specific delivery strategies, e.g., intraparenchymal and intrathecal infusions, are available and capable of delivering drugs directly to the brain parenchyma or cerebrospinal fluid (CSF) for pain management, these options are very invasive and not always practical [7,8,9], and generally not accepted by patients. One non-invasive strategy to allow efficient brain delivery of polypeptides is intranasal (IN) administration. Peptides delivered by the IN route are absorbed into the mucus membrane of the nasal cavity and reach both the brain and the systemic blood circulation [10]. Moreover, peptides can be specifically formulated for IN delivery to improve bioavailability in the brain [10]. Similarly, devices have been developed for the purpose of improving nose-to-brain delivery [11,12,13,14]. Intranasal delivery has tremendous potential to allow brain delivery of therapeutic polypeptides that are otherwise impossible to deliver.

Pain is a major public clinical concern with significant social and economic impact worldwide [15]. Of all chronic conditions, pain is the most disabling and has the most negative impact on quality of life [15]. Usually, acute pain conditions are well managed [16]. However, chronic pain conditions including migraine, trigeminal neuralgia, neuropathic and orthopedic pain conditions are especially difficult to treat [17,18,19,20]. Chronic pain is associated with altered activity in multiple networks in the central nervous system (CNS) [21,22]. In addition, chronic pain may result in changes in afferent inputs to the brain, brain structure and modulatory pathways [22,23,24]. Therefore, analgesics for many chronic pain conditions need to reach the peripheral and/or central nervous system at sufficient concentrations for effective treatment.

While recognizing the wide range of chronic pain conditions that could be included, in this narrative review we focus on IN delivery of therapeutic polypeptides for chronic pain associated with the trigeminal system, including headache, migraine and trigeminal neuralgia and other chronic craniofacial pain conditions. We first focus on the studies demonstrating that polypeptides reach the CSF and brain tissue and briefly discuss the nasal–cerebral pathway. Next, we review our studies and the relevant recent literature on IN delivery of oxytocin for trigeminal and chronic pain. Lastly, we review the literature on other IN applied polypeptides that work as analgesics.

## 2. IN Polypeptides Reach the CSF and Brain

Multiple studies show that polypeptides such as oxytocin reach the trigeminal nerve, cerebrospinal fluid (CSF) and the brain after IN delivery. Specifically, our and other groups [25,26,27] using a radiolabeling approach in rodent models have shown that IN applied radiolabeled oxytocin accumulates in the respiratory and olfactory epithelium, trigeminal ganglion, olfactory bulb, and brain regions such as the thalamus, hypothalamus, midbrain and pons (Table 1). In addition, recent studies using rodents and non-human primates provide direct evidence that IN delivery of labeled oxytocin reaches the brain via olfactory and/or trigeminal pathways, depositing in target tissues, including the amygdala and hippocampus [28,29]. IN administration of exogenous polypeptides such as oxytocin has been shown to result in functionally relevant increases in CSF concentrations [30,31]. A recent study by Lee et al. provides direct evidence for substantial penetrance of IN administered labeled oxytocin into the CSF in non-human primates [30]. Additionally, CSF samples measured before and after IN administration of oxytocin in pigs show oxytocin levels in CSF sufficient to influence neural activity [31]. These studies provide evidence that intranasally applied polypeptides can reach the nerves and brain regions involved in pain pathogenesis.

Polypeptides administered subcutaneously or intravenously have very short half-lives (3–5 min for oxytocin) likely due to rapid intravascular catabolism, renal elimination and/or degradation in the liver [32,33]. In addition, only tiny fractions of peripherally applied polypeptide reach the CNS (approximately 0.002% for oxytocin) [32,33]. By contrast, IN application of polypeptides leads to much higher brain concentrations; for some peptides, more than 95% is directly transported from the nasal cavity into the CNS [33]. Thus, neural and physiological effects of polypeptides can sometimes be observed after IN delivery and not after intravenous injections [33,34]. However, a recent study provides evidence that a high dose of continuous intravenous infusion (with consistently high plasmatic concentration) of oxytocin was able to induce changes in regional cerebral blood flow in the amygdala, a region rich in oxytocin receptors [35]. One possible explanation for this discrepancy is that oxytocin may reach hypothalamic sites of partial BBB leakiness, allowing access to oxytocinergic cells which, through a positive feedback, cause an increase in brain oxytocin [14]. However, high peripheral oxytocin concentrations can potentially lead to unforeseen side effects via peripheral oxytocin or vasopressin receptor activation [36].

Multiple studies show that IN delivery of some polypeptides, including oxytocin, produce analgesic effects, at least in part due to their effects on CNS pain circuitry. For example, our group, using electrophysiological and immediate-early gene expression experiments in rodents, showed that IN oxytocin can drastically inhibit responses to craniofacial painful stimulation in a specific brainstem region (trigeminal nucleus caudalis (TNC) [26]. In addition, our group provided evidence that IN oxytocin greatly reduces the number of activated neurons in the TNC in a rodent migraine model [26]. Similarly, IN application of neuropeptide S has been reported to inhibit arthritis pain-related behaviors via changes in amygdalar activity [37]. Consistent with these animal studies, a human study showed that oxytocin specifically modulates neural processes contributing to pain perception [38]. This study observed an association between the analgesic effect of oxytocin and oxytocin-induced modulation of cortical activity after noxious stimulation [38]. These studies clearly show that IN administration of polypeptides such as oxytocin not only reaches the brain but has significant effect on a variety of pain conditions.

## 3. Nasal–Cerebral Mechanism

**Figure 1:**
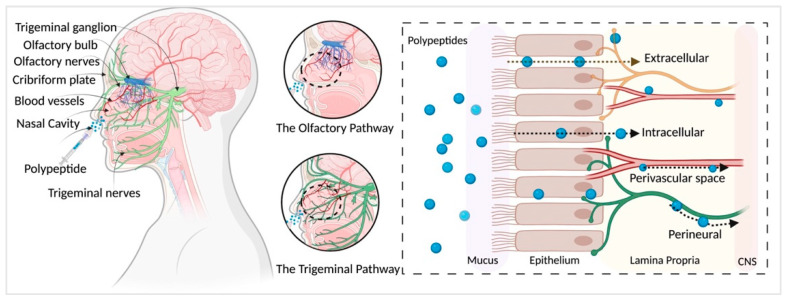
Pathways of polypeptide distribution after IN administration. Olfactory and trigeminal pathways are major contributors to intranasal delivery of polypeptides to the nervous system. Compounds pass through the nasal epithelium to reach the lamina propria largely via extracellular transport and reach the CNS mainly via bulk flow through the perineural, perivascular spaces. Created with BioRender.com (accessed on 21 May 2021).

The transport of IN administered polypeptides into the brain is not completely understood. Studies show that the olfactory nerve pathway, trigeminal nerve pathway, vascular and perivascular space, CSF and lymphatic systems may all play a role. We have briefly explained some of these pathways (Figure 1); for an in-depth review, see Dhuria et al. [10] and Lochhead et al. [39].

Nasal Anatomy: Nasal hairs, mainly the nasal mucosa, represent an efficient first line of defense for the body’s airways. The vestibular, olfactory and respiratory zones make up the nasal cavity. The vestibular area located immediately at the nostril openings is lightly vascularized, comprising non-ciliated epithelial cells with nasal hairs. The absorption of drugs in this region is minimal due to the small surface area (~0.6 cm^2^).

The respiratory region, which occupies the largest part of the nasal cavity (~130 cm^2^), is highly vascularized and may thus serve as an efficient absorption surface for topically applied drugs. The respiratory zone consists of mucus-producing goblet cells (20%) and ciliated cells (80%) and the cells are connected via tight junctions. These cells together perform a cleansing mechanism by trapping and transporting particulates in the mucus, termed as mucociliary clearance (MCC). The MCC is approximately 20 min and has thus become an important consideration for effective intranasal drug delivery. In addition, the trigeminal nerve innervates the respiratory epithelium in the nasal passage, suggesting a key role in the IN transport of compounds to the brain [40,41].

The olfactory region is in the deep upper part of the nasal cavity under the cribriform plate that has high perforations providing access to the CNS. The olfactory region corresponds to ~10% of the total surface area of the nasal cavity (~15 cm^2^) and is highly vascularized. The olfactory epithelium is innervated by both the olfactory and trigeminal nerves. The passage of compounds from the nose to the brain via the olfactory zone might occur by various pathways/mechanisms, as discussed below.

Previous radiolabeled tracer studies [42,43,44,45] using polypeptides and proteins provide evidence that the olfactory and trigeminal nerve pathways are major contributors to intranasal delivery. Intranasally administered compounds first cross the surface of the nasal epithelium and reach the lamina propria, located under the basement membrane of the epithelial surface [10,39]. The lamina propria contains components of the olfactory nerves and the trigeminal nerves that provide the anatomical connections between the nasal passage to the CNS.

Compounds have been shown to be rapidly transported from the nasal passages to the olfactory bulb via extracellular pathways [39,46]. Extracellular transport likely involves diffusion along peripheral olfactory or trigeminal nerves [10,39]. By contrast, intracellular pathway mechanisms are shown to be a slow process and are not likely to provide a significant mode of transport for IN compounds [10,39]. In addition to extracellular and intracellular transport, IN compounds are shown to distribute through the perineural spaces of the olfactory and trigeminal nerve bundles, via bulk flow [47,48].

There are vascular connections between the nasal passages and the brain that provide a potential mode of transport for IN compounds [10,41,49]. For example, there are blood vessel connections between the cribriform plate and nasal lamina propria [10,41,49]. Also, the nasal–olfactory artery sends branches from the olfactory bulb into the lamina propria [10,41,49]. Although not clearly understood, the perivascular spaces of these blood vessels are considered a potential extracellular pathway to enter the brain [39,50,51,52]. After reaching the brain, compounds can be distributed throughout the CNS via bulk flow mechanisms and/or more rapidly via the perivascular spaces [39,53]. For example, IN studies using [^125^I]-labeled IGF-1 show rapid distribution towards the CNS in about 30 min [42]. Such rapid distribution is thought to occur due to extracellular convection rather than diffusion or intracellular transport. One hypothesis for the fast extracellular transport is via bulk flow in the perivascular spaces in the nose-to-brain pathway [42,46].

## 4. Analgesic Effects of Oxytocin

IN administered oxytocin has been investigated by several groups for relief of migraine and other pain types [26]. For example, analgesic effects of IN applied oxytocin have also been shown for pain after mild traumatic brain injury [54], wound pain [55], chronic low back pain [56] and chronic pelvic pain [57]. Modulation of neuronal activity of the trigeminal nerve, limbic and cortical brain regions as well as ascending and descending pain pathways in the spinal cord have been suggested as potential mechanisms for oxytocin’s pain-modulating effects [58]. For example, a recent study in chronic low back pain patients using functional magnetic resonance imaging suggests that striatum plays a key role in the underlying pain-modulating effects of oxytocin in patients [59]. In addition to chronic back pain, oxytocin plays an analgesic role in migraine. Recently, Garcia-Boll and colleagues have demonstrated that oxytocin reduces trigeminocervical complex neuronal firing evoked by meningeal electrical stimulation, a well-established electrophysiological model of migraine [60]. Other potential mechanisms of migraine relief include blockade of CGRP release, which plays an important role in migraine. For instance, intranasal treatment with oxytocin has been shown to decrease the frequency of headaches in both chronic and high-frequency episodic migraineurs [26]. IN oxytocin has been studied in highly standardized experimental pain protocols. For example, Paloyelis et al. demonstrated that IN oxytocin reduced subjective pain ratings and attenuation of the amplitude of N1, N2 and P2 components in a double-blind, placebo-controlled cross-over study in healthy volunteers using laser-evoked potentials [38].

Interestingly, sex-specific effects of intranasal oxytocin on pain perception have been observed. For example, Tracy et al. showed that intranasal oxytocin increased the perceived intensity of noxious heat stimuli in women with chronic neck and shoulder pain, but not in men [61]. Similarly, a recent study on the perception of wound pain showed that intranasal oxytocin reduced wound pain in men, but not in women [55]. These studies on sex-specific effects suggest that oxytocin and endogenous sex hormones may interact to influence pain perception. Clinical studies using IN oxytocin for pain is summarized in Table 2.

**Figure 2:**
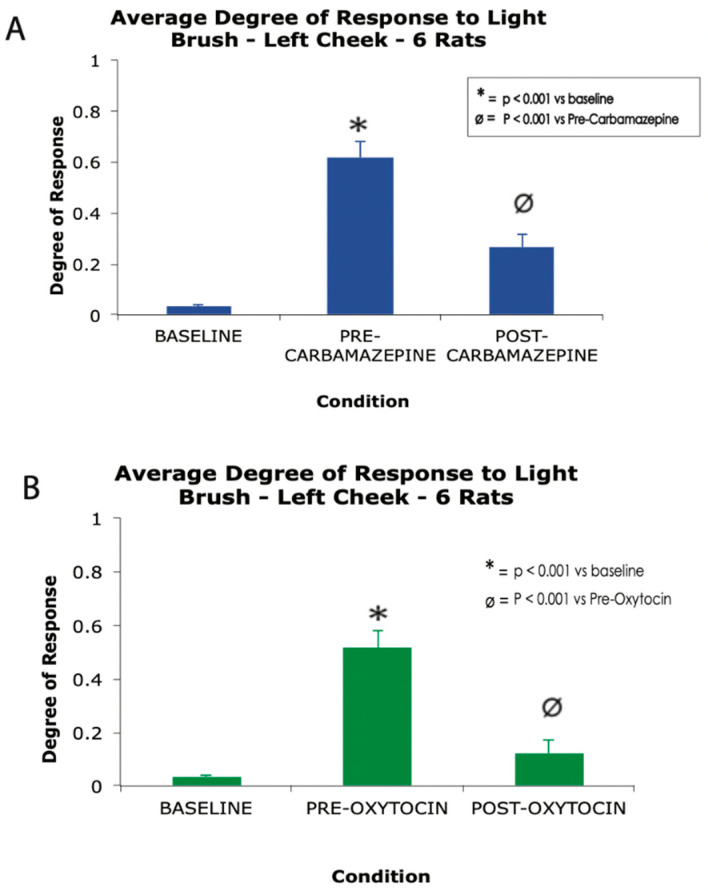
A high dose carbamazepine (**A**) and single dose of nasal oxytocin (**B**) produce robust analgesia in a rat model of trigeminal neuralgia. * *p* < 0.001 vs. baseline, Ø *p* < 0.001 vs. oxytocin, one-way ANOVA. Error bars represent standard error of the mean.

Previously, Tzabazis et al. have shown that nasally applied oxytocin concentrates predominantly in the trigeminal nerve, ganglia and nucleus, as well as the dura mater—a key terminal field for the trigeminal nerve—and have shown that nasal oxytocin inhibits the firing of peripheral and central trigeminal nociceptive neurons [26]. In a recent study presented here for the first time, the authors demonstrate the analgesic effect of intranasal oxytocin in a rat model of trigeminal neuralgia. In this study, polymer crystals were stereotaxically applied between the trigeminal nerve root and the crista petrosal bone in order to produce chronic compression of the nerve root [62], which results in a behavioral phenotype highly reminiscent of human trigeminal neuralgia, including exquisite peri-oral hypersensitivity to brush and grimacing. Results from this original study showed that daily treatment with carbamazepine for 4 days or a single dose of IN oxytocin significantly decrease responsiveness to brush stimulation of the peri-oral face (Figure 2).

Overall, preclinical and clinical studies demonstrate that intranasal delivery of oxytocin works well as an analgesic for craniofacial pain. However, future studies investigating the general clinical utility of this treatment as well as more precise explorations into the mechanism of delivery and mechanisms of analgesic action are warranted.

## 5. Other Polypeptides That Work as Analgesics

Intranasally administered polypeptides have been investigated by several groups for potential analgesic effects. Candidate compounds include but are not limited to oxytocin, vasopressin, desmopressin, calcitonin, enkephalins, dermorphin analogue, insulin, neuropeptide S, conotoxins and others. Clinical studies using IN polypeptides for pain is summarized in Table 2.

There are several publications that have investigated the analgesic effect of intranasally administered desmopressin in patients with acute renal colic. Constantinides et al. [63] reported complete resolution of colic pain 30 min after IN application of desmopressin in 54% of patients. Several other groups reported similar analgesic effects of desmopressin alone or in combination with diclofenac [64], tramadol [65] or ketorolac [66]. Although the analgesic effects of desmopressin were, in general, lower compared to systemically applied traditional analgesic drugs, most studies concluded that the ease of administration and the favorable side-effect profile of IN desmopressin make it an interesting option for selected patients.

Intranasally administered calcitonin has been used as an analgesic in a variety of patient populations, including patients with McCune-Albright syndrome-associated bone pain [67]. This treatment has also been successful with other bone-associated pain, such as the pain associated with distal radial fractures [68] and vertebral crush fractures, especially when osteoporosis-related [69]. Beneficial effects have also been postulated for trigeminal neuralgia [70], complex regional pain syndrome [71] and other difficult-to-treat pain syndromes [72,73].

Enkephalins are endogenous opioid pentapeptides, binding to both µ- and δ-opioid receptors, which are found in high concentrations in the brain. The intranasal delivery route has been postulated to allow for bypassing the BBB and hence yield maximum concentrations in the target areas, producing robust analgesia while limiting systemic side-effects such as constipation. Our group has investigated the analgesic effects of intranasal administration of a herpes-based viral vector encoding for human proenkephalin in a rodent model of traumatic brain injury (TBI) [74]. Two days after inducing mild TBI, rats received either the vector encoding for human proenkephalin (SHPE) or a control vector encoding for lacZ (SHZ.1). Control vector-treated rats developed facial allodynia post TBI, but those treated with the enkephalin vector did not. This effect lasted for at least 45 days, which was the latest time point investigated. Following intranasal administration of the viral vectors, robust expression of human proenkephalin was demonstrated in the trigeminal ganglia of rats treated with SHPE, but not after SHZ.1 treatment. Another group [75] has shown that intranasal administration of enkephalins yields an analgesic effect in rodent pain models and that the analgesic effects could be enhanced by co-administration of enzyme inhibitors and/or absorption enhancers to reduce rapid destruction by extracellular peptidases. Another interesting approach is to design enkephalin derivatives that are more resistant against these peptidases and extend their half-lives [76].

A relatively new development is intranasal application of conotoxin derivatives to alleviate pain. Clinical use of omega-conotoxin MVIIA (ziconotide) is severely limited by its poor ability to cross the BBB and hence needs to be administered intrathecally. However, robust analgesic effects have been reported for both IN administered ziconotide [77] and a biochemically modified version of the cone snail peptide [78].

**Table 2:** Summary of clinical trials using intranasal polypeptides for pain.

Pain Model	Polypeptide	Outcome	References
Chronic pelvic pain	Intranasal oxytocin	Twice-daily administration of oxytocin may represent an adjuvant analgesic for refractory pelvic pain	[57]
Thermal pain perception	Intranasal oxytocin	Sex-specific effects of intranasal oxytocin on thermal pain perception, suggesting that oxytocin and endogenous sex hormones may interact to influence noxious stimuli	[61]
Migraine	Intranasal oxytocin	Intranasal treatment with oxytocin decreases the frequency of headaches in both chronic and high-frequency episodic migraineurs	[26]
Colic pain	Intranasal desmopressin	Complete resolution of colic pain 30 min after IN application of desmopressin in 54% of patients	[63]

## 6. Therapeutic Considerations and Delivery Devices

The nasal anatomy and physiology including nasal mucosa, MCC, humidity and airflow may influence the intranasal administration. In addition, factors such as lipophilicity, molecular weight, dose per spray puff, volume per spray puff, pH and osmolality of the compound all play a role in optimal internasal delivery.

Strategies to improve drug uptake and prolong resistance/stability is an important consideration for optimal nasal deposition and delivery. Mucoadhesive polymers such as chitosan and polyacrylic acid have been used as excipients for intranasal formulations [79]. These polymers interact with the mucins to prolong residence of the drug in the mucosa and thus improve drug uptake [80]. Mucoadhesive polymers can also modify the trajectory of the formulations to reach the nasal cavity and thus reduce drug loss [81]. In addition, preservatives such as lipophilic chlorobutanol help prolong the stability of the nasal drug formulation [79]. Dose volume of the sprays is also related to the nasal deposition of the formulation. Generally, the dose volume on the market is 50 μL to 100 μL [82,83] and volumes larger than 100 μL are known to run down the posterior pharynx [83].

In addition, the spray pattern, droplet size distribution and viscosity of the formulation all influence the nasal deposition and thus the delivery to the brain. Nasal spray pattern is largely influenced by the formulation of the compound, and it is speculated that a narrow plume angle might enable the spray to penetrate deeper into the nasal cavity and result in large deposition area [82]. In addition, droplet size influences nasal deposition, where larger droplets tend to deposit at the anterior area, whereas smaller droplets deposit in the inner area of the nasal cavity [82]. Physiological properties such as viscosity of the formulation influence the droplet size of the nasal spray. Results from Gua et al.’s study suggest that low-viscosity formulations (producing smaller droplets) significantly enhance middle and posterior coverage of the nasal cavity compared to higher viscosity formulations [84].

For efficient nose-to-brain delivery, intranasally administered compounds should reach the olfactory region [85]. In this context, significant efforts are made to optimize polypeptide delivery via nose-to-brain transport by enhancing drug distribution and absorption through the olfactory epithelium. For example, a breath-powered device has been used to deliver low-dose oxytocin and has been reported to enhance deposition in the intranasal sites for direct nose-to-brain delivery [34,86]. In addition, the Precision Olfactory Delivery (POD^®^) device targets the delivery of drugs into the upper nasal cavity operated by pressure [87]. Furthermore, therapeutic strategies to incorporate polypeptides into a vehicle system that provides prolonged drug stability and supports optimal drug delivery need to be considered. For example, liposomes, nanoparticles and micelles have recently gained potential as useful tools for targeting the brain with reduced toxicity in nasal mucosa and the CNS [88,89].

## 7. Limitations of Intranasal Delivery

Many compounds that are useful to treat chronic pain are limited by their transport to the brain due to the BBB. As reviewed here, IN delivery provides a non-invasive strategy to deliver polypeptides to the brain. Nasal–olfactory and trigeminal pathways are reliable pathways to deliver compounds to the brain for chronic pain while minimizing side effects.

There are some limitations of the nose–brain delivery method. For example, the volume of the compound that can be IN administered is relatively small (~100 μL). In addition, the surface area of the olfactory epithelium critical for nose–brain delivery, short retention time for drug absorption and the influence of mucosal secretion all limit the drug delivery to the brain. Furthermore, the limitations of nasal delivery of liquid formulations and long-term use of compounds are the limited microbiological stability and the presence of preservatives, which may lead to irritation and allergic effects [90]. Indication of nasal congestion due to cold or allergies may interfere with this method of delivery. Pumps with a shorter tip to avoid contact with sensitive mucosal surfaces and side actuation have been designed to aid during allergic conditions [91]. Overall, strategies to combat these limitations are constantly developing and remain critical for the development of new nasal delivery devices.

## 8. Future Directions

Future directions for intranasal pain management include identifying and investigating potential drug candidates, improving delivery strategies and optimizing central nervous target concentrations. Drug candidates for (co-)analgesia using an intranasal administration route under investigation include NK1-receptor antagonists [92], ketamine [93] and esketamine [94], nalbuphine [95], ketorolac [96], dexmedetomidine [97] and many more. There is also a wide variety of research on permeation-enhancing agents, mucolytic agents, muco-adhesive agents, in situ gelling agents and enzyme-inhibiting agents in the formulation of nasal drug delivery systems [98]. In addition, nanoemulsions [99] and liposomal formulations [89] have also been used for intranasal drug delivery. In general, all of these pharmacological modifications and approaches intend to standardize and optimize drug delivery across the BBB into the CNS.

## 9. Conclusions

Chronic pain is difficult to treat, in part because it is associated with altered activity in multiple networks and changes in the pain pathways in the peripheral and central nervous systems. IN delivery is a proven strategy to allow targeted delivery of polypeptides to the trigeminal nerve and ganglia as well as pain-associated brain sites for the treatment of pain. After IN administration, radiolabeled oxytocin has been shown to be preferentially deposited in the trigeminal system and is also present in the hippocampus, thalamus, midbrain and pons, key regions in pain processing. The results described in this review demonstrate that there is overwhelming evidence for peripheral and central nervous system effects due to intranasally applied polypeptides. While it is not completely understood how these peptides are deposited into the peripheral and central nervous systems, it has become clear that nasal application of polypeptides has tremendous potential to provide analgesia in conditions where systemic application is impossible or has significant limitations.

## Figures and Tables

**Table 1:** Uptake of radiolabeled oxytocin after intranasal administration in rats. Intranasal I-125-oxytocin is initially concentrated in the respiratory and olfactory epithelium. Labeled oxytocin is preferentially taken up by the trigeminal system and is also present in the hippocampus, thalamus midbrain and pons, key regions in the pain processing pathway. Note that a relatively high value in the blood is likely reflective of oxytocin fragments as the compound is rapidly degraded in the blood.

Tissue	Mean (nM) ± SE
Respiratory epithelium	731,147 ± 76,889
Olfactory epithelium	19,348 ± 8141
Trigeminal ganglion	574 ± 181
Trigeminal maxillary N.	471 ± 117
Trigeminal mandibular N.	676 ± 235
Trigeminal ophthalmic N.	424 ± 235
Dorsal dura	152 ± 11.6
Ventral dura	271 ± 43.4
Spinal dura	31 ± 7.9
Olfactory bulbs	33 ± 13
Ant. olfactory nucleus	34 ± 10
Caudate-putamen	39 ± 10
Septal nucleus	24 ± 6
Parietal cortex	29 ± 6
Hippocampus	15 ± 3
Thalamus	21 ± 4
Hypothalamus	21 ± 4
Midbrain	23 ± 12
Pons	26 ± 11
Cerebellum	20 ± 8
Blood	63 ± 4

## Data Availability

Data supporting the reported results can be found in the Synapse public database: https://www.synapse.org, accessed on 1 June 2021.
